# Inactivation of highly transmissible livestock and avian viruses including influenza A and Newcastle disease virus for molecular diagnostics

**DOI:** 10.3389/fvets.2024.1304022

**Published:** 2024-03-07

**Authors:** Jennifer L. Welch, Ram Shrestha, Heather Hutchings, Narinder Pal, Randall Levings, Suelee Robbe-Austerman, Rachel Palinski, Karthik K. Shanmuganatham

**Affiliations:** National Veterinary Services Laboratories, Veterinary Services, Animal and Plant Health Inspection Service, United States Department of Agriculture, Ames, IA, United States

**Keywords:** animal virus, molecular diagnostics, inactivation, influenza virus, molecular transport media

## Abstract

There is a critical need for an inactivation method that completely inactivates pathogens at the time of sample collection while maintaining the nucleic acid quality required for diagnostic PCR testing. This inactivation method is required to alleviate concerns about transmission potential, minimize shipping complications and cost, and enable testing in lower containment laboratories, thereby enhancing disease diagnostics through improved turn-around time. This study evaluated a panel of 10 surrogate viruses that represent highly pathogenic animal diseases. These results showed that a commercial PrimeStore® molecular transport media (PSMTM) completely inactivated all viruses tested by >99.99%, as determined by infectivity and serial passage assays. However, the detection of viral nucleic acid by qRT-PCR was comparable in PSMTM and control-treated conditions. These results were consistent when viruses were evaluated in the presence of biological material such as sera and cloacal swabs to mimic diagnostic sample conditions for non-avian and avian viruses, respectively. The results of this study may be utilized by diagnostic testing laboratories for highly pathogenic agents affecting animal and human populations. These results may be used to revise guidance for select agent diagnostic testing and the shipment of infectious substances.

## Introduction

Prevention efforts are in place to protect animals in the United States from highly contagious diseases caused by classical swine fever virus (CSFV), African swine fever virus (ASFV), foot-and-mouth disease virus (FMDV), eastern equine encephalitis virus (EEEV), Newcastle disease virus (NDV), and highly pathogenic avian influenza A virus (HPAI), among others (1–3). When an approved vaccine or treatment is unavailable, depopulation becomes the mandated course of action for affected farms (4). This issue underscores the critical importance of prevention efforts, as depopulation disrupts the food supply and has major financial implications for producers and consumers (1). For example, the largest ASF outbreak to date was reported in China between 2018 and 2019, resulting in estimated total financial losses of more than USD$111.2 billion (5). The ongoing HPAI outbreak, first identified in the United States in 2022, has led to current US economic losses estimated at a range of USD $2.5–3 billion (6). In addition, some of these viruses, including FMDV, EEEV, NDV, and HPAI, are zoonotic, making them a concern for public health (7–10). Prevention efforts include active surveillance testing (11).

Active surveillance testing requires the shipment of suspected samples of highly contagious diseases to BSL3+ high containment facilities for PCR confirmatory testing to meet select agent requirements (12, 13). The shipment of potentially infectious substances is costly and time-sensitive and it requires specialized shipping containers and a cold chain to avoid the deterioration of samples for PCR testing (14). An inactivation method that protects the nucleic acid content essential for PCR testing while removing the threat of transmission and minimizing the temperature requirement is urgently needed to minimize shipping costs and derestrict confirmatory testing to lower biosafety-level facilities (BSL2). Implementation of an inactivation method will alleviate the strain that the high demand for PCR testing is placing on limited BSL3 facilities across the United States, thereby decreasing testing response time and improving practical difficulties. This includes improving the complications of sharing reagents among international laboratories (15). The accidental shipment of live anthrax to laboratories within the United States due to incomplete inactivation highlights the importance of an inactivation method that provides a safety buffer for use with high-consequence pathogens (16).

PrimeStore® molecular transport media (PSMTM) tubes contain proprietary reagents that inactivate both viruses and bacteria by disrupting lipid membranes and inhibiting replication machinery while stabilizing nucleic acids (PrimeStore^®^, Longhorn Vaccines and Diagnostics) (17, 18). As indicated by the publicly available safety data sheet, this reagent contains a mixture of guanidine thiocyanate, ethanol, and n-lauroylsarcosine (19). Guanidine thiocyanate-based reagents have previously been shown to effectively inactivate poliovirus and FMDV, though the comparison of results should be interpreted with caution as the exact formulation of PSMTM is proprietary (20, 21). Previous studies have shown that ethanol and n-lauroylsarcosine inactivate various enveloped and some non-enveloped viruses by interacting with lipids and denaturing proteins (22).

PSMTM is authorized by the Food and Drug Administration (FDA) as a Class II device for the collection of samples suspected of containing influenza A virus (IAV) and *Mycobacterium tuberculosis* (17). Other studies have shown that PSMTM effectively inactivates SARS-CoV-2, adenovirus type 5, influenza A H3N2, and HPAI H5N1 at ambient temperature (17, 23). Influenza A H1N1 studies showed that viral RNA was preserved in PSMTM for 30 days at 25°C, and cycle threshold (CT) values were minimally reduced from days 0 to 30 (23). In addition, the PSMTM tubes were utilized during the COVID-19 pandemic for SARS-CoV-2 clinical testing (17, 24). Studies showed that a 10-min contact time was sufficient to completely inactivate SARS-CoV-2 and no virus was detected in tittering assays or after serial passage on susceptible cells (24). Together, these data support that PSMTM inactivates viral pathogens and preserves nucleic acids at ambient temperature, thereby permitting normal shipping procedures without disrupting normal PCR testing workflows.

In this study, we assess the effectiveness of PSMTM in the inactivation of surrogate viruses that represent USDA Veterinary Services (*VS*) select agent viruses, some of which are considered foreign animal disease agents, including ASFV, CSFV, and FMDV (Table 1) (3). Surrogate viruses were utilized in this study, as inactivation testing was completed in BSL2 conditions. ASFV is the only member of the Asfarviridae family, making surrogate selection a challenge. To address this, we utilized the species-relevant swinepox virus and the genetically similar vaccinia virus (32–34). Both swinepox and vaccinia virus are members of the Poxvirus family and, such as Asfarviridae, are nucleocytoplasmic large DNA viruses (NCLDV) that share a common ancestor (35). Surrogate selections for other target viruses were previously identified by others and included bovine viral diarrhea virus (BVDV) as a surrogate for CSFV and senecavirus A (SVA) as a surrogate for FMDV (36, 37). In addition, less pathogenic strains of target viruses were used as surrogates when possible, such as low pathogenic avian influenza (LPAI) H7N3, LPAI H6N1, and LPAI H5N3 as surrogates for HPAI, lentogenic NDV as a surrogate for velogenic NDV, swine influenza virus (SIV) as a surrogate for IAV, and EEEV/Sindbis virus chimera as a surrogate for EEEV (38). Therefore, surrogate virus selections were best matched to the target viruses for NDV, HPAI, and IAV (same species, genus, and family), followed by EEEV (chimera of target virus and same genus), CSF (same genus and family), FMDV (same family), and ASF (common ancestor).

**Table 1:** Surrogate virus characteristics and specifics used for PSMTM inactivation studies.

Target virus	Target virus characteristics and genome size	Surrogate virus	Surrogate virus strain	Surrogate virus characteristics and genome size	Surrogate propagation	Surrogate titer/passage	Surrogate PCR reference
African swine fever virus	dsDNA; enveloped; 170-190 kb	Vaccinia	Modified vaccinia ankara (MVA)	dsDNA; enveloped; 190 kb	Primary CEF^a^	Vero76/Secondary CEF	(25)
		Swinepox	Primary isolate	dsDNA; enveloped; 175 kb	PK-15^b^	PK-15/PK-15	(26)
Classical swine fever virus	+ssRNA; enveloped; 12.3 kb	Bovine viral diarrhea virus 1a (CPE)	Singer	+ssRNA; enveloped; 12.5 kb	MDBK^c^	MDBK/MDBK	(27)
Highly pathogenic avian influenza	-ssRNA (segmented); enveloped; 13.5 kb	Low pathogenic avian influenza	H7N3 A/turkey/Utah/22–014130-002/2022	-ssRNA (segmented); enveloped; 13.5 kb	Embryonated eggs	MDCK/MDCK	(28)
		Low pathogenic avian influenza	H6N1 A/Turkey/South Dakota/21–036406-002/2021	-ssRNA (segmented); enveloped; 13.5 kb	Embryonated eggs	MDCK/MDCK	(28)
		Low pathogenic avian influenza	H5N3 A/Turkey/Minnesota/21–035506-007/2021	-ssRNA (segmented); enveloped; 13.5 kb	Embryonated eggs	MDCK/MDCK	(28)
Velogenic Newcastle disease virus	-ssRNA; enveloped, 15 kb	Lentogenic Newcastle disease virus	LaSota	-ssRNA; enveloped, 15 kb	Embryonated eggs	Vero/Secondary CEF	(29)
Influenza A virus	-ssRNA (segmented); enveloped; 13.5 kb	Swine influenza virus	H3N2 A/SW/TX/1/98	-ssRNA (segmented); enveloped; 13.5 kb	MDCK^d^	MDCK/MDCK	(28)
Eastern equine encephalitis virus (EEEV)	+ssRNA; enveloped; 11 kb	EEEV/Sindbis virus chimera	North American	+ssRNA; enveloped; 13.7 kb	Vero	Vero/Vero	(30)
Foot and mouth disease virus	+ssRNA; non-enveloped; 8.3 kb	Senecavirus A	USA/MO15-029085/2015	+ssRNA; non-enveloped; 7.2 kb	PK-15	PK-15/PK-15	(31)

a CEF: chicken embryo fibroblast.

b PK: porcine kidney.

c MDBK: Madin–Darby bovine kidney.

d MDCK: Madin–Darby canine kidney.

Several other studies have evaluated commercial transport media for their compatibility with nucleic acid testing (39). These studies have shown that PSMTM and other commercially available transport media inactivate several avian viruses as well as SARS-CoV-2 (14, 17, 23, 39–43). However, nucleic acid preservation results were not available in all these studies. There is a paucity of thoroughly evaluated commercial transport media that inactivate viruses in multiple families while protecting the viral nucleic acid for subsequent use in diagnostic testing. The selected surrogate viruses used in this study represent viruses with diverse classifications in the exterior structure (envelope vs. non-enveloped), nucleic acid composition (DNA vs. RNA), and nucleic acid structure (single vs. double strand) (Table 1). At least one representative virus was utilized for each target virus and selected based on ease of ability to grow to high titers. Diagnostic samples are collected from biological material that may influence inactivation efficacy. Previous studies identified that the presence of blood may interfere with the viricidal activity of disinfectants (44); however, other studies have shown that viruses remain sensitive to chemical inactivation in the presence of blood (45). Therefore, due to the high pathogenicity and transmission consequences of these viruses, it is important to validate PSMTM with not only viruses from different families but also multiple sample types and collection conditions, such as the volume of PSMTM and incubation time. In this study, the PCR detection and inactivation of virus replication were evaluated following the PSMTM treatment. In addition, inactivation was also evaluated for sera and cloacal swabs.

## Materials and methods

### Viruses

EEEV/Sindbis virus chimera, swinepox, SVA, LPAI H6N1, LPAI H7N3, LPAI H5N3, SIV H3N2, lentogenic NDV, and BVDV 1a were provided by the USDA Diagnostic Virology Laboratory (DVL). The vaccinia virus was purchased from the American Type Culture Collection (ATCC). Viruses were propagated and tittered by median tissue culture infectious dose (TCID_50_) in appropriate egg or cell systems (Table 1), and all eggs and cells used for propagation and tittering were provided by the USDA Diagnostic Bioanalytical and Reagents Laboratory (DBRL) Proficiency Testing and Reagents (PTR) section. The use of animal products was approved by the National Veterinary Services Laboratories/Center for Veterinary Biologics Institutional Animal Care and Use Committee (APH-22-1033). The study was conducted in accordance with local legislation and institutional requirements. Viruses were propagated in minimum essential media (MEM) with 5% fetal bovine serum, 4 mM L-glutamine (Gibco), 0.015 g/L of penicillin, 0.1 g/L of streptomycin, and 1x non-essential amino acids (Gibco). Virus titrations were performed in MEM with 2% fetal bovine serum and the same supplements as listed for the propagations.

### Virus natural decay inactivation

200 uL of virus was added to each well of a 24 well tissue culture plate in triplicate for each timepoint. Viruses were incubated at ambient temperature until the indicated timepoint. To mitigate the effects of evaporation, viruses were collected at the indicated timepoint by adding 200 μL of virus propagation media to each well and performing repeated pipetting. The collected viruses were then frozen once at -80°C until titration by TCID_50_ on susceptible cells (Table 1).

### Virus PSMTM inactivation

Viruses were treated with PrimeStore® molecular transport media (PSMTM) according to manufacturer instructions. One part virus was combined with three parts PSMTM and incubated for 1 h at ambient temperature. Virus infectivity (TCID_50_) was compared to no-inactivation control, where phosphate-buffered saline (PBS) replaced PSMTM and was treated the same. To mimic the relevance of animal diagnostic samples collected from biological material, viruses were combined with species-appropriate serum or swabs. Viruses combined with serum were as follows: SIV, vaccinia, swinepox, EEEV/Sindbis virus chimera, SVA, and BVDV. Viruses combined with swabs were as follows: LPAI H7N3, LPAI H6N1, LPAI H5N3, and NDV. For sera studies, the virus was diluted 1:1 with swine or bovine serum prior to treatment with PSMTM or PBS. For swab studies, each virus in liquid media was added to a tube containing a cloacal swab collected from an uninfected chicken and then incubated at ambient temperature for approximately 10 min. The virus in liquid media was then collected by pipette and treated with PSMTM or PBS control, as described above. All control and inactivation procedures were completed in three experimental replicates. Three parts PSMTM or PBS control to one part virus ratios were maintained for all experiments except for the varying ratio experiments. Ratio variations of 1 part PSMTM or PBS control to 1 part virus, 1 part PSMTM or PBS control to 3 parts virus, 1 part PSMTM or PBS control to 10 parts virus, and 1 part PSMTM or PBS control to 100 parts virus were also evaluated with EEEV/Sindbis virus chimera to assess PSMTM inactivation if the recommended ratio is not maintained. All TCID_50_ results were completed using 10-fold serial dilutions.

### Virus serial passage

PSMTM or PBS control-treated viruses were passaged on susceptible cells (Table 1) for a minimum of three serial passages. PSMTM is highly cytotoxic to cells (Supplementary Figure S1). To minimize the cytotoxicity of PSMTM, buffer exchange was completed prior to the first passage of PSMTM and PBS control-treated viruses. PSMTM and PBS control-treated virus material were diluted to a ratio of 1:10 in MEM without supplements. Diluted material was then added to an Amicon® 50 kDa cutoff centrifugal filter (Millipore) and centrifuged at 1500 x *g* until the diluted material was concentrated to the original volume (approximately 10 min). This filter cutoff was chosen as all viruses included in this study were larger than 50 kDa and would be retained in the filter reservoir. The concentrated material was then diluted to a ratio of 1:10 in MEM, and 50 kDa concentration steps were repeated. After final concentration to the original volume, PSMTM- and PBS-treated viruses were diluted to a ratio of 1:10 in MEM for virus propagation, and the total volume (2.0 mL) was added to one well of a 6-well plate. Cells were incubated with PSMTM- or PBS-treated viruses until cytopathic effect (CPE) was observed in PBS control-treated virus wells or for a maximum of 7 days. Passaged products were collected by three freeze/thaw cycles of cells and supernatant together. Passaged products were then centrifuged at 1500 x *g* for 10 min to remove cell debris. A fraction (10%) of the clarified product was diluted in a ratio of 1:10 in MEM for virus propagation, and the total volume (2.0 mL) was added to naive cells (one well of a 6-well plate) for each subsequent passage. The collection of PSMTM-inactivated or control-treated viruses from susceptible cells was designated as a passaged virus.

**Figure 1:**
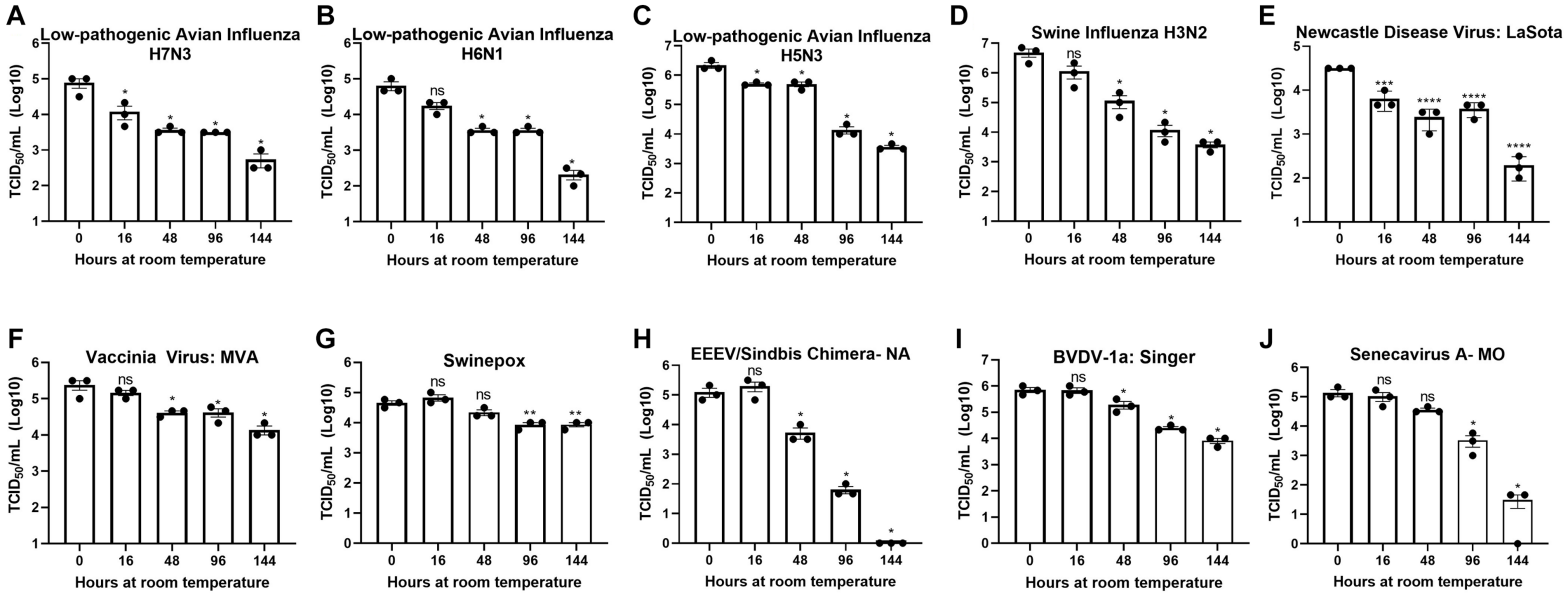
Diverse viruses are stable at ambient temperature. Virus recovery over time deposited on a plastic surface at ambient temperature for **(A)** low pathogenic avian influenza virus (LPAI) H7N3; **(B)** LPAI H6N1; **(C)** LPAI H5N3; **(D)** swine influenza virus (SIV) H3N2; **(E)** Newcastle disease virus (NDV); **(F)** vaccinia; **(G)** swinepox; **(H)** eastern equine encephalitis virus (EEEV)/Sindbis chimera; **(I)** bovine viral diarrhea virus (BVDV); and **(J)** Senecavirus A. Virus recovery was assessed by TCID_50_ titer using the cell line described in Table 1. Each timepoint was compared to timepoint 0. Significance was determined using Student’s *t*-test. ^*^ *p* < 0.05; ^**^ *p* < 0.01; ^***^ *p* < 0.001; ^****^ *p* < 0.0001; ns, not significant. Error bars represent the standard error of the mean (SEM) of triplicate experiments.

**Figure 2:**
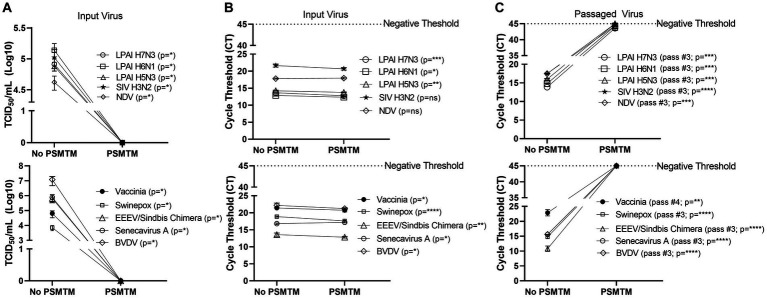
PSMTM effectively inactivates diverse animal viruses while maintaining the detection of nucleic acid. **(A)** Virus recovery after PrimeStore® molecular transport media (PSMTM) inactivation or PBS no-inactivation control. Virus recovery was assessed by TCID_50_ titer using the cell line described in Table 1. **(B)** Nucleic acid cycle threshold (CT) detection after PSMTM inactivation or PBS no-inactivation control. **(C)** Serial passage endpoint nucleic acid cycle threshold (CT) detection after PSMTM inactivation or PBS no-inactivation. The serial passage was completed using the cell line in Table 1 and the description outlined in Methods. Nucleic acid was detected according to the reference in Table 1 and the description in the Methods section. PSMTM-treated viruses were compared to the corresponding no-PSMTM control, and inactivation was assessed using the manufacturer’s recommended conditions. Significance was determined using Student’s t-test. ^*^ *p* < 0.05; ^**^ *p* < 0.01; ^***^ *p* < 0.001; ^****^ *p* < 0.0001; ns, not significant. Error bars represent the standard error of the mean (SEM) of triplicate experiments. Low pathogenic avian influenza virus (LPAI); swine influenza virus (SIV); Newcastle disease virus (NDV); eastern equine encephalitis virus (EEEV); and bovine viral diarrhea virus (BVDV).

**Figure 3:**
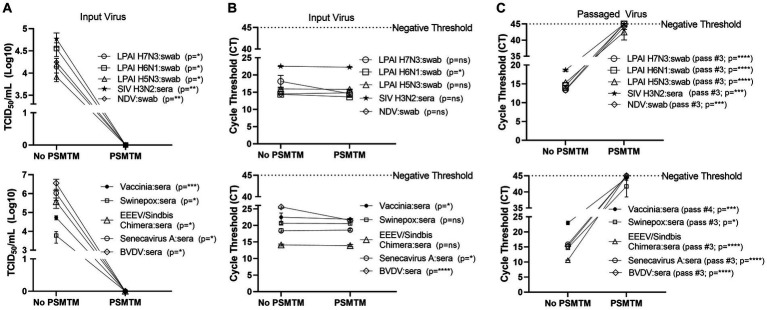
Presence of biological material does not affect the effectiveness of PSMTM on the inactivation or nucleic acid detection of diverse viruses. Viruses were combined with species-appropriate sera or cloacal swabs as described in the Methods section. **(A)** Virus recovery after PrimeStore® molecular transport media (PSMTM) inactivation or PBS no-inactivation control. Virus recovery was assessed by TCID_50_ titer using the cell line described in Table 1. **(B)** Nucleic acid cycle threshold (CT) detection after PSMTM inactivation or PBS no-inactivation control. **(C)** Serial passage endpoint nucleic acid cycle threshold (CT) detection after PSMTM inactivation or PBS no-inactivation. Serial passage was completed using the cell line in Table 1 and the description outlined in the Methods section. Nucleic acid was detected according to the reference in Table 1 and the description in Methods. PSMTM-treated viruses were compared to the corresponding no-PSMTM control, and inactivation was assessed using the manufacturer’s recommended conditions. Significance was determined using Student’s *t*-test. ^*^ *p* < 0.05; ^**^ *p* < 0.01; ^***^ *p* < 0.001; ^****^ *p* < 0.0001; ns, not significant. Error bars represent the standard error of the mean (SEM) of triplicate experiments. Low pathogenic avian influenza virus (LPAI); swine influenza virus (SIV); Newcastle disease virus (NDV); eastern equine encephalitis virus (EEEV); and bovine viral diarrhea virus (BVDV).

### Virus PCR detection

Nucleic acid was extracted from 200 uL of virus material treated with PSMTM or PBS, as well as from each passage product from the serial passages described above. Nucleic acid was extracted using the MagMax™ CORE nucleic acid purification kit (Applied Biosystems) on a KingFisher Flex (Thermo Scientific) instrument with the provided MagMax_CORE_Flex script (with heat) according to manufacturer instructions. Nucleic acid was detected by a virus-specific primer/probe according to the indicated reference sequences (Table 1), utilizing the TaqMan™ Fast Virus 1-Step Master Mix (Applied Biosystems) and QuantStudio™ 5 (Thermo Scientific) real-time PCR instrument. The cycling conditions for LPAI H6N1, LPAI H7N3, LPAI H5N3, SIV H3N2, NDV, and BVDV were as follows: 50°C for 5 min, 95°C for 20 s, followed by 45 cycles of 95°C for 15 s and 60°C for 45 s. Cycling conditions for the vaccinia virus were as follows: 95°C for 20 s, followed by 45 cycles of 95°C for 10 s and 60°C for 30 s. The cycling conditions for EEEV/Sindbis virus chimera and SVA were as follows: 50°C for 15 min, 95°C for 2 min, followed by 45 cycles of 95°C for 15 s and 60°C for 30 s. Cycling conditions for swinepox were as follows: 50°C for 5 min, 95°C for 20 s, followed by 45 cycles of 95°C for 15 s and 53°C for 1 min. Data were analyzed utilizing Design and Analysis software (version 2.6, Thermo Scientific), where threshold values were set at 0.100. A CT of 45 was used as the no amplification negative threshold. The comparison of stock virus TCID_50_ and CT values revealed similar trends, validating assay parameters (Supplementary Figure S2). All control and inactivation procedures were completed in three experimental replicates, and each PCR was completed with three technical replicates.

### Virus PCR stability

200 μL of PSMTM or PBS control-treated viruses were added to screw-cap tubes in triplicate for each timepoint. Screw-cap tubes were utilized to mimic PSMTM manufacturer tube conditions that may be utilized in field sample collection. Timepoints were collected for a maximum of 21 days to mimic the maximum anticipated transit time once a field sample is collected until it reaches a laboratory for diagnostic testing. PSMTM or PBS control-treated viruses were incubated at ambient temperature until the indicated timepoint, where viruses were collected by adding 200 μL of virus propagation media to each tube and performing repeated pipetting. The collected viruses were frozen once at -80°C until titration by TCID_50_ on susceptible cells (Table 1), and PCR detection was performed as described above.

### Cell viability

Cell viability was determined by an MTT (Invitrogen) assay, where the cell lines utilized in this study were plated in 96-well plates and incubated with PSMTM or PBS control dilutions. PSMTM or PBS were initially diluted to the manufacturer’s recommended ratio used for PSMTM inactivation studies described above, where virus propagation media (not containing viruses) were substituted for one part virus to assess the effect of PSMTM alone (without viruses) on cell viability, as these viruses induce CPE and would confound the assessment of the effect of PSMTM on cell viability. PSMTM or PBS control-treated media were then serially diluted 1:10 and incubated with cells for 48 h. Cells were then incubated with 5 mg/mL of MTT reagent in the dark for 3.5 h at 37°C before the addition of 4 mM HCl in isopropanol and monolayer disruption by pipetting. Absorbance was measured at 490 nm using a BioTek plate reader. PSMTM cell viability values were normalized to an equivalent PBS dilution, set at 100%.

### Statistics

Statistical significance was determined by GraphPad software v9.3.1 (GraphPad Prism 9) using a two-tailed Student’s *t*-test. *p-*values of <0.05 were considered significant. Error bars represent the standard error of the mean (SEM) from three biological replicates.

## Results

### Diverse viruses show similar patterns of stability at ambient temperature

To assess the potential risk of shipping transmissible high-containment samples without an inactivation method applied at the time of sample collection, we evaluated the natural decay of the viruses used in this study over time. Virus infectivity at each timepoint was compared to timepoint 0. Timepoint 0 shows that a minimum of 4-logs of virus were deposited and recovered for all viruses tested (Figures 1A–J).

**Figure 4:**
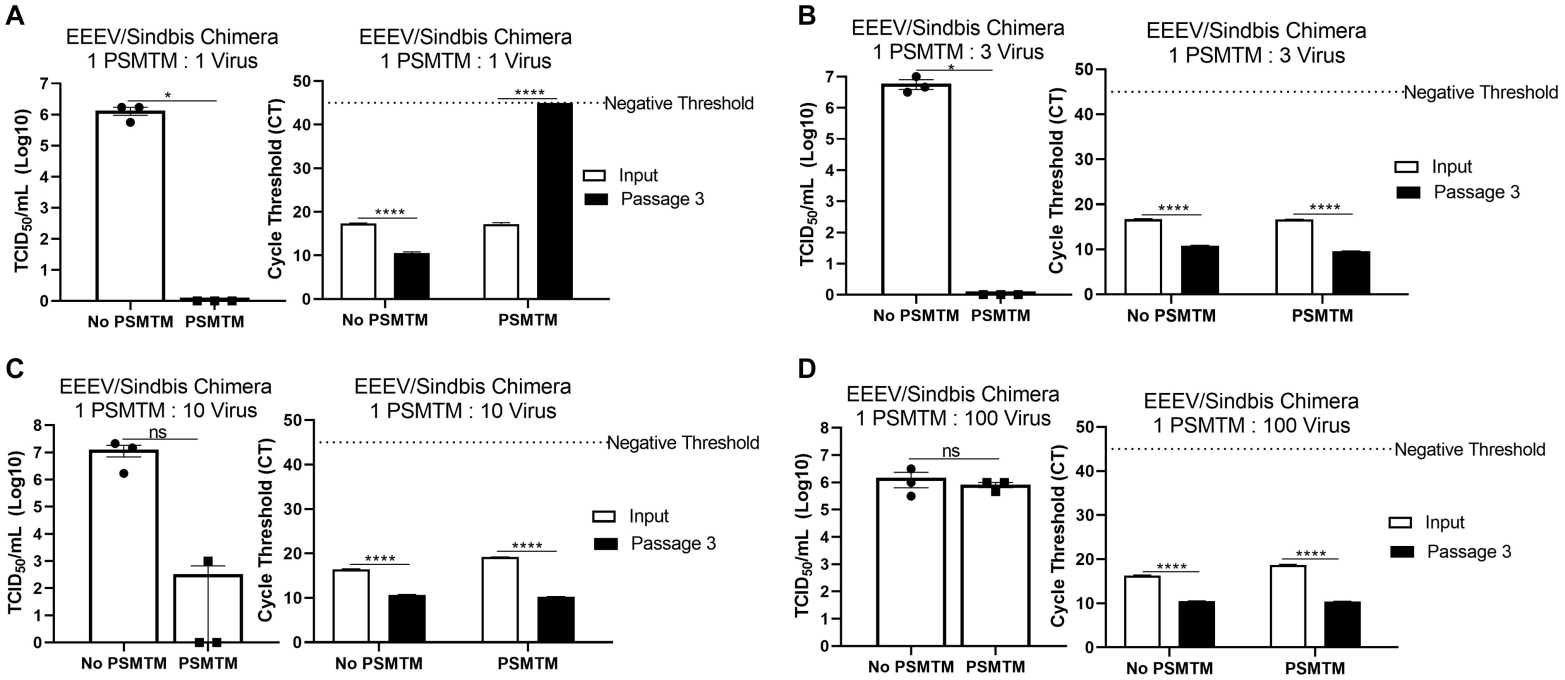
PSMTM to virus ratio determines the effectiveness of inactivation. Virus recovery and nucleic acid detection were determined after PrimeStore® molecular transport media (PSMTM) inactivation or PBS no-inactivation control utilizing various treatment to virus ratio conditions: **(A)** 1:1 ratio; **(B)** 1:3 ratio; **(C)** 1:10 ratio; and **(D)** 1:100 ratio. Virus recovery was assessed by TCID_50_ titer using the cell line described in Table 1. Nucleic acid was detected according to the reference in Table 1 and the description in the Methods section. PSMTM-treated viruses were compared to the corresponding no-PSMTM control. Significance was determined using Student’s *t*-test. ^*^ *p* < 0.05; ^**^ *p* < 0.01; ^***^ *p* < 0.001; ^****^ *p* < 0.0001; ns, not significant. Error bars represent the standard error of the mean (SEM) of triplicate experiments. Eastern equine encephalitis virus (EEEV).

Although natural decay varied among individual viruses, results show a maximum reduction of 1-log after 48 h at ambient temperature and survival of all viruses after 144 h at ambient temperature, with the exception of EEEV/Sindbis virus chimera, which was completely inactivated at 144 h (Figures 1A–J). These data indicate that highly pathogenic viruses may survive prolonged periods of time under natural conditions and demonstrate the need for an inactivation method that can be utilized at the time of sample collection to reduce the risk of transmission.

### PSMTM completely inactivates viruses representing highly contagious animal diseases

Viruses were treated with PSMTM according to the manufacturer’s recommended conditions or PBS (no-PSMTM) for control comparison. Viral replication was assessed by TCID_50_ and susceptible cell serial passage, and nucleic acid was evaluated by qRT-PCR. PSMTM treatment completely inactivated all viruses tested compared to PBS control. No infectivity persisted in PSMTM-treated viruses, and virus titers were reduced >4-log (99.99%) (Figure 2A; Table 2). Despite no remaining virus infectivity after PSMTM treatment, CT values of viruses treated with PSMTM compared to no-PSMTM control were highly comparable and varied by less than 1.2 CT values (Figure 2B; Table 2). A CT value change of 3.3 is considered to represent a 1-log (10-fold) change (46). To ensure complete inactivation, PSMTM- and control-treated viruses were serially passaged on susceptible cells (Table 1). CT values of PSMTM-treated viruses reached a negative threshold with no observable cell infectivity by passage endpoint of a minimum of three passages, indicating no nucleic acid amplification or virus replication (Figure 2C; Supplementary Figures S3, S4). Conversely, CT values of control-treated viruses were stable or showed reduced CT (increased nucleic acid) at the passage endpoint compared to input (Figure 2C; Supplementary Figure S3). Cell infectivity was also observed at the passage endpoint for control-treated viruses (Supplementary Figure S3). Together, these data show a complete inactivation of PSMTM-treated virus replication without deterioration of the nucleic acid that is needed for PCR testing.

**Table 2:** Log10 TCID_50_^a^ reduction and qRT-PCR cycle threshold (CT) change in PSMTM^b^ treatment vs. control treatment viruses.

Virus	Strain	Log_10_ reduction (PSMTM vs. control treatment)	CT change (PSMTM vs. control treatment)
Vaccinia	Modified Vaccinia Ankara (MVA)	>4.5	0.7
Swinepox	Primary isolate	>3.5	1.2
Bovine viral diarrhea virus 1a	Singer	>7.0	0.9
Low pathogenic avian influenza	H7N3 A/Turkey/Utah/22–014130-002/2022	>4.5	1.1
Low pathogenic avian influenza	H6N1 A/Turkey/South Dakota/21–036406-002/2021	>5.0	0.5
Low pathogenic avian influenza	H5N3 A/Turkey/Minnesota/21–035506-007/2021	>4.5	1.1
Newcastle disease	LaSota	>4.0	0.3
Swine influenza	H3N2 A/SW/TX/1/98	>5.0	0.5
EEEV/Sindbis virus chimera	North American	>5.5	0.8
Senecavirus A	USA/MO15-029085/2015	>5.5	0.1

a TCID50, Median tissue culture infectious dose.

b PSMTM, PrimeStore® molecular transport media.

We assessed the PSMTM inactivation of viruses in the presence of species-relevant sera or, in the case of avian surrogates, cloacal swabs. Consistent with our findings without added biological materials, PSMTM treatment completely inactivated all virus-spiked sera or virus-spiked cloacal swabs (Figure 3A). CT values of virus-spiked sera or virus-spiked cloacal swabs treated with PSMTM were comparable to those of the no-PSMTM control (Figure 3B). Importantly, serial passage of PSMTM-treated viruses in the presence of serum or a swab approached the negative threshold by passage endpoint, whereas control-treated viruses did not (Figure 3C; Supplementary Figure S3). These data show that the presence of biological material does not affect the PSMTM inactivation of viruses.

### Maintenance of appropriate PSMTM ratio is required for effective virus inactivation

The collection of diagnostic samples may not always achieve the exact 3 PSMTM:1 virus inactivation ratio recommended by the manufacturer. To assess the safety of PSMTM inactivation use if the recommended ratio is not maintained, we evaluated virus infectivity and serial passage of viruses treated with lesser amounts of PSMTM. EEEV/Sindbis virus chimera was evaluated due to its high titer growth. The results show that 1 PSMTM: 1 virus ratio completely inactivated virus infectivity >6-log and virus serial passage reached a negative threshold by passage endpoint, whereas the control-treated virus did not achieve similar results (Figure 4A). However, although the 1 PSMTM: 3 virus ratio inactivated virus infectivity >6-log, virus serial passage results revealed that the virus was not completely inactivated, where PSMTM-treated virus CT values were comparable to control-treated virus CT values by passage endpoint (Figure 4B). Results of 1 PSMTM: 10 virus and 1 PSMTM: 100 virus ratio alterations further showed that virus was not inactivated as measured by infectivity and serial passage assays (Figures 4C,D). These results indicate that minimal variations in the PSMTM inactivation ratio recommended by the manufacturer may still completely inactivate the virus. However, caution should be used during sample collection to maintain the appropriate ratio to ensure complete inactivation.

### PSMTM may modestly enhance the stability of viral nucleic acid content required for PCR testing

During the shipment of samples, which may take multiple days to reach laboratories for diagnostic testing, nucleic acid may deteriorate or environmental conditions such as evaporation may influence inactivation efficacy (47). We evaluated the effect of PSMTM on inactivation stability and virus nucleic acid detection over time. LPAI H7N3 and vaccinia virus were evaluated due to their moderate and high stability, respectively, of infectivity over time at ambient temperature (Figures 1A,F). PSMTM-treated or control-treated viruses were deposited into screw-cap tubes to mimic sample collection tubes and collected at the indicated timepoint. Virus infectivity for each timepoint was compared to timepoint 0 of each respective treatment.

**Figure 5:**
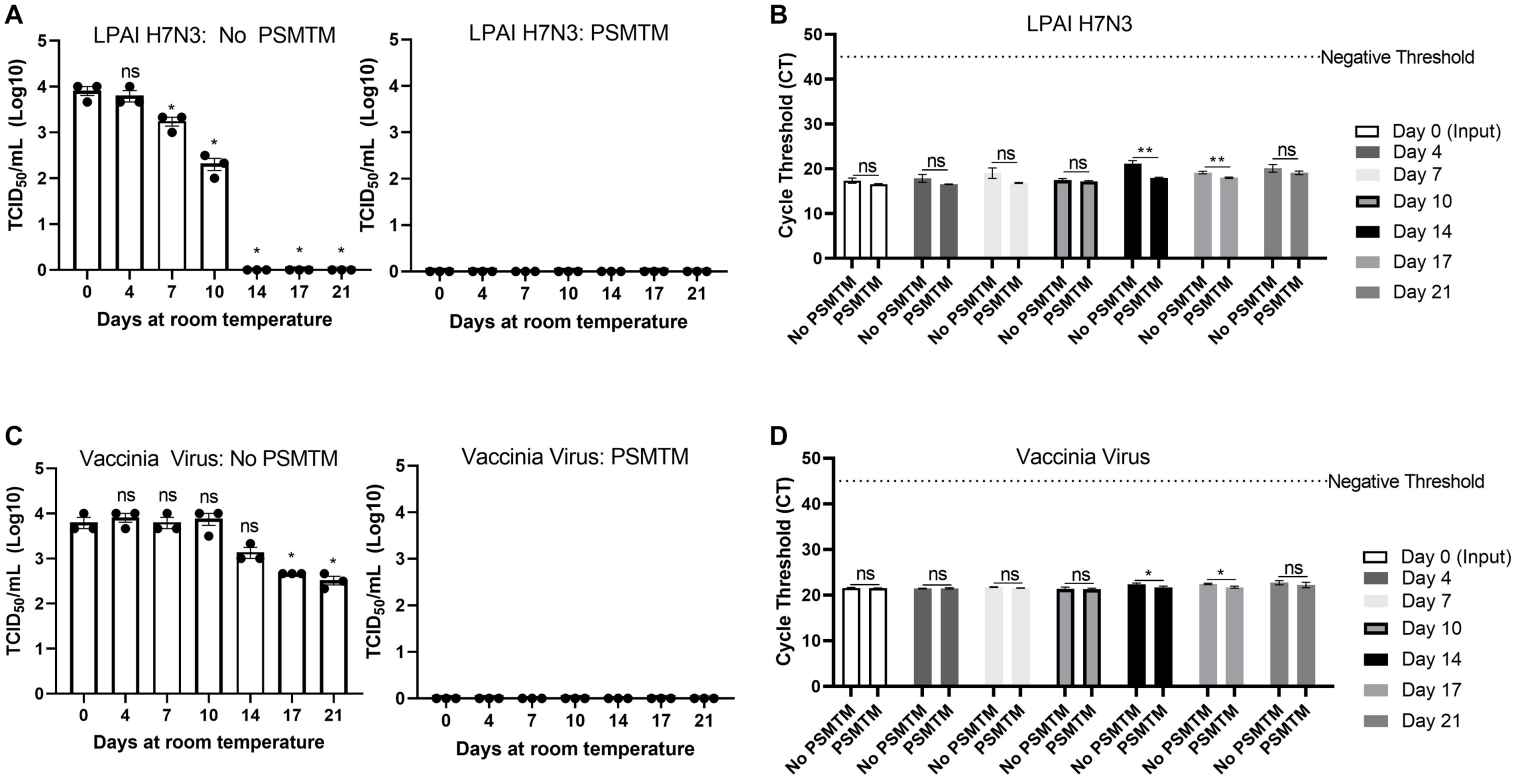
Virus inactivation and nucleic acid detection are stable over time in PSMTM. Virus recovery and nucleic acid detection over 21 days for viruses deposited in a plastic screw-cap tube at ambient temperature. Inactivation was assessed using the manufacturer’s recommended conditions. **(A)** Virus recovery was assessed by TCID_50_ for PBS no-inactivation control-treated LPAI H7N3 (left) and PrimeStore® molecular transport media (PSMTM)-treated LPAI H7N3 (right). **(B)** Virus nucleic acid cycle threshold (CT) detection for PBS no-inactivation control and PSMTM-treated LPAI H7N3. **(C)** Virus recovery was assessed by TCID_50_ for PBS no-inactivation control-treated vaccinia virus (left) and PSMTM-treated vaccinia virus (right). **(D)** Virus nucleic acid cycle threshold (CT) detection for PBS no-inactivation control and PSMTM-treated vaccinia virus. **(A,C)** TCID_50_ titer was determined in the appropriate cell line as identified in Table 1. Each timepoint was compared to timepoint 0 for each respective treatment. **(B,D)** Nucleic acid was detected according to the reference in Table 1 and the description in Methods. PSMTM-treated viruses were compared to the corresponding no-PSMTM control. Significance was determined using Student’s *t*-test. ^*^ *p* < 0.05; ^**^ *p* < 0.01; ns, not significant. Error bars represent the standard error of the mean (SEM) of triplicate experiments. Low pathogenic avian influenza virus (LPAI).

Infectivity results show that PSMTM-treated viruses were completely inactivated at all timepoints compared to control-treated viruses (Figures 5A,C). These data indicate that PSMTM inactivation is complete and virus infectivity has not recovered over time. Control-treated virus infectivity results reveal similar trends to our natural decay findings (Figures 1A,F), where LPAI H7N3 was moderately stable until complete loss of infectivity occurred by day 14 (Figure 5A). However, LPAI H7N3 nucleic acid was detected over 21 days despite the loss of infectivity (Figures 5A,B). The comparison of PSMTM- and control-treated LPAI H7N3 viruses shows comparable CT values at each timepoint with only a moderate increase in CT stability (reduced CT value or increased nucleic acid) in PSMTM at later timepoints (Figure 5B). The control-treated vaccinia virus was highly stable, and infectivity was only reduced <2-logs by 21 days at ambient temperature (Figure 5C). Vaccinia virus nucleic acid was detected over 21 days, and CT values were comparable for PSMTM- and control-treated viruses, with a slight increase in CT stability (reduced CT value or increased nucleic acid) in PSMTM at later timepoints (Figure 5D). These data indicate that PSMTM may enhance the stability of viral nucleic acid at later timepoints, although the effect is modest.

## Discussion

Highly pathogenic disease outbreaks, including the 2022 US HPAI outbreak and the COVID-19 pandemic, strain the diagnostic capacity of already limited high containment facilities. There is a need for a reagent that protects the nucleic acid required for diagnostic testing while also inactivating pathogens to permit testing in lower containment facilities. PSMTM was previously utilized during the COVID-19 pandemic for diagnostic RT-PCR testing (24) and is authorized by the FDA for the collection of IAV and *Mycobacterium tuberculosis* samples (17). However, additional studies are needed to determine the inactivation of a diverse range of viruses with a paired assessment of the viral nucleic acid needed for testing. In this study, we evaluated the inactivation of diverse animal virus surrogates, including four influenza A viruses, through titering and serial passage assays and assessed nucleic acid preservation through qRT-PCR. This is one of the largest studies completed to date that includes animal viruses of high consequence from different families. This inactivation reagent is one of the only reagents available for veterinary diagnostic samples that eliminates the need for temperature control, which may increase surveillance testing.

The viruses used in this study showed reduced but detectable replication after several days at ambient temperature in liquid media (Figure 1). Although conditions may considerably vary between studies, these virus decay trends are generally in agreement with those reported by others (48–55). Therefore, an inactivation reagent utilized at the time of sample collection is ideal to alleviate concerns about transmission during shipping procedures and facilitate testing at lower containment facilities. All 10 viruses tested in this study, including four influenza A viruses of avian and swine origin, were completely inactivated (≥99.99% reduction) by PSMTM when utilized according to manufacturer recommendations (Figure 2). Serial passage of PSMTM-treated viruses revealed no replicating virus (Figure 2). Inactivation was not dependent on virus titer, as the virus titers used in this study varied from 4- to 7-log/mL (Figures 2, 3). These titers are biologically relevant as animal model studies showed titers of ≤5log plaque forming units per mL (PFU/mL) for FMDV (56), 6–8log median hemadsorbing dose per milliliter (HAD_50_/mL) for ASF (57), 5–7log TCID_50_/mL for CSF (58), ≤7log PFU per gram tissue for EEEV (59) and IAV (60), and ≤ 7log and ≤ 8log TCID per gram tissue for NDV (61) and HPAI (62), respectively. Inactivation results were verified in the presence of biological material (virus-spiked sera or virus-spiked cloacal swabs) (Figure 3) and are consistent with findings by others, demonstrating that chemical inactivation methods are not sensitive to the presence of biological material (45). However, natural infection studies are needed to confirm that PSMTM inactivation is not sensitive to saturating concentrations of biological material.

Previous studies have shown that PSMTM inactivation is complete even with reduced contact time from the manufacturer’s recommended duration of 60 min, where a contact time of as little as 2 min was identified as sufficient (63). However, previous studies did not evaluate the importance of the volumetric ratio of inactivation media to the virus-containing sample volume. Variation of the inactivation media ratio in our results showed that inactivation was incomplete with a modest deviation from the manufacturer’s recommended ratio of 3:1 (Figure 4). Titering and serial passage results revealed that inactivation was complete when the ratio was altered from the manufacturer’s recommended ratio of 3:1 to 1:1; however, inactivation was incomplete with an additional decrease in the ratio of the inactivation media to virus sample media (Figure 4). Incomplete inactivation was not attributed to a significant increase in the titer of virus sample media, as the PBS without PSMTM inactivation control conditions remained within 1-log for all ratio deviations (Figure 4). While previous studies indicated that the contact time can be reduced without compromising inactivation, these results indicate that adherence to the manufacturer’s recommended volumetric ratio provides a reliable safety margin to ensure complete inactivation.

Importantly, nucleic acid CT values were within 1.5 for all viruses in PSMTM and PBS no-inactivation control conditions, indicating the preservation of the nucleic acids despite the inactivation of virus replication (Figures 2, 3). Other studies have shown that CT values were comparable for the detection of human respiratory viruses collected in PSMTM compared to other media (23, 64). Together, the results of this study and those from previous studies by others show that PSMTM maintains the stability of both viral RNA and viral DNA for human and animal viruses despite the reduced environmental stability of viral RNA (23, 64, 65). In addition, results from this study show that inactivation and nucleic acid stability appear to be maintained over extended periods of time. The incubation of LPAI H7N3 and vaccinia virus over 21 days revealed that PSMTM inactivation was complete with no observation of virus replication, despite comparable or modestly enhanced nucleic acid detection for PSMTM compared to PBS no-inactivation control (Figure 5). The endpoint of this study was 21 days; however, other studies showed that nucleic acid was detected from samples in PSMTM up to 196 days following sample collection (64).

In this study, PSMTM was completely effective at inactivating all viruses tested. Our results are consistent with others that evaluated the use of PSMTM with human viruses (23, 64). However, the viruses used in this study were surrogate viruses, and additional studies are required with the target viruses represented in this study to validate inactivation with actual select agent viruses. Our data indicate that the use of this viral transport media, according to the manufacturer’s recommended procedures, successfully inactivates all the viruses tested, and alternative procedures, including differing sample types, would require further evaluation to ensure inactivation. This inactivation method may improve testing turn-around time during highly pathogenic disease outbreaks by de-restricting diagnostic testing. This method may also be used to ensure that sample quality is maintained during normal shipping procedures. The results of this study may be applied to other pathogens that require molecular testing and may be sensitive to environmental conditions such as temperature and time to testing, and in scenarios where samples may be collected in field sites with limited resources. However, molecular testing was only evaluated in this study by qRT-PCR, and other molecular-based methods would require additional validation.

## Data availability statement

The original contributions presented in the study are included in the article/Supplementary material, further inquiries can be directed to the corresponding author.

## Ethics statement

The animal study was approved by National Veterinary Services Laboratories/Center for Veterinary Biologics Institutional Animal Care and Use Committee APH-22-1033. The study was conducted in accordance with the local legislation and institutional requirements.

## Author contributions

JW: Conceptualization, Writing – original draft, Writing – review & editing, Investigation. RS: Writing – review & editing, Investigation. HH: Writing – review & editing, Resources. NP: Writing – review & editing, Investigation. RL: Writing – review & editing, Conceptualization. SR-A: Writing – review & editing, Conceptualization. RP: Writing – original draft, Writing – review & editing. KS: Conceptualization, Writing – original draft, Writing – review & editing, Supervision.
